# Resolving the cellular specificity of TSPO imaging in a rat model of peripherally-induced neuroinflammation

**DOI:** 10.1016/j.bbi.2021.05.025

**Published:** 2021-08

**Authors:** Marta Vicente-Rodríguez, Nisha Singh, Federico Turkheimer, Alba Peris-Yague, Karen Randall, Mattia Veronese, Camilla Simmons, Abdul Karim Haji-Dheere, Jayanta Bordoloi, Kerstin Sander, Ramla O. Awais, Erik Årstad, NIMA Consortium, Diana Cash, Christine A. Parker

**Affiliations:** a Department of Neuroimaging, Institute of Psychiatry, Psychology & Neuroscience, King's College London, London, United Kingdom; b GlaxoSmithKline, Stevenage, London, United Kingdom; c PET Centre, St Thomas' Hospital, London, United Kingdom; d The Wellcome Trust Consortium for the Neuroimmunology of Mood Disorders and Alzheimer's Disease (NIMA), United Kingdom; e Centre for Radiopharmaceutical Chemistry, University College London, London WC1E 6BS, United Kingdom; f School of Biomedical Engineering and Imaging Sciences, King’s College London, London SE1 7EH, United Kingdom

**Keywords:** Astrocytes, Biomarkers, DPA-714, Microglia, Macrophages, Neurons, Neuroinflammation, TSPO, TSPO, 18kDa translocator protein, PET, positron emission tomography, LPS, lipopolysaccharide, ic. LPS, intracerebral administered LPS, CNS, central nervous system, TBI, traumatic brain injury, OSEM, ordered subset expectation maximization, AUC, area under the curve, TACs, time-activity curves, SUV, standardized uptake values, ROIs, regions of interest, PFA, paraformaldehyde, DAB, diaminobenzidine, TSPO+, TSPO positive cells, Iba1+, Iba1 positive cells, TSPO+Iba1+, double positive cells for TSPO and Iba1, TSPO+Iba1-, positive cells for TSPO and negative for Iba1. *Tmem119*, transmembrane protein 119, *Ccr2*, C–C Motif Chemokine Receptor 2

## Abstract

The increased expression of 18 kDa Translocator protein (TSPO) is one of the few available biomarkers of neuroinflammation that can be assessed in humans *in vivo* by positron emission tomography (PET). TSPO PET imaging of the central nervous system (CNS) has been widely undertaken, but to date no clear consensus has been reached about its utility in brain disorders. One reason for this could be because the interpretation of TSPO PET signal remains challenging, given the cellular heterogeneity and ubiquity of TSPO in the brain.

The aim of the current study was to ascertain if TSPO PET imaging can be used to detect neuroinflammation induced by a peripheral treatment with a low dose of the endotoxin, lipopolysaccharide (LPS), in a rat model (ip LPS), and investigate the origin of TSPO signal changes in terms of their cellular sources and regional distribution. An initial pilot study utilising both [^18^F]DPA-714 and [^11^C]PK11195 TSPO radiotracers demonstrated [^18^F]DPA-714 to exhibit a significantly higher lesion-related signal in the intracerebral LPS rat model (ic LPS) than [^11^C]PK11195. Subsequently, [^18^F]DPA-714 was selected for use in the ip LPS study.

Twenty-four hours after ip LPS, there was an increased uptake of [^18^F]DPA-714 across the whole brain. Further analyses of regions of interest, using immunohistochemistry and RNAscope Multiplex fluorescence V2 *in situ* hybridization technology, showed TSPO expression in microglia, monocyte derived-macrophages, astrocytes, neurons and endothelial cells. The expression of TSPO was significantly increased after ip LPS in a region-dependent manner: with increased microglia, monocyte-derived macrophages and astrocytes in the substantia nigra, in contrast to the hippocampus where TSPO was mostly confined to microglia and astrocytes. In summary, our data demonstrate the robust detection of peripherally-induced neuroinflammation in the CNS utilising the TSPO PET radiotracer, [^18^F]DPA-714, and importantly, confirm that the resultant increase in TSPO signal increase arises mostly from a combination of microglia, astrocytes and monocyte-derived macrophages.

## Highlights

• [^18^F]DPA-714 PET showed an increased brain signal following peripheral LPS challenge.
• The cellular origin of TSPO signal appears to depend on the brain region examined.
• TSPO signal increase in the hippocampus arises from microglia and astrocytes.
• Microglia, macrophages and astrocytes are the main contributors in the substantia nigra.
• Macrophages and microglial cells expressing TSPO are distinguished by RNAscope.

## 1 Introduction

Neuroinflammation, the activation of inflammatory pathways in the brain and central nervous system (CNS), plays a complicit role in several neurological and psychiatric disorders, and prolonged activation of microglia, the resident immune cells in the brain, is likely associated with pathological progression (Guzman-Martinez et al., 2019). The proinflammatory role of microglia has been widely documented in the past decade due to the availability of imaging biomarkers designed to detect *in vivo* brain inflammation. Most of these biomarkers belong to a family of positron emission tomography (PET) radiotracers that bind to an 18kD Translocator protein (TSPO) located on the outer mitochondrial membrane of various cells, with a relatively high expression in microglia (Turkheimer et al., 2015). Studies using TSPO PET radiotracers have shown increased TSPO binding, corresponding to increased neuroinflammation, in the brain of patients with Alzheimers disease (AD), Parkinson disease (PD), stroke, traumatic brain injury (TBI), and multiple sclerosis (MS), amongst others (Dimitrova-Shumkovska et al., 2020, Nutma et al., 2019). Altered TSPO binding has also been associated with psychiatric disorders such as depression and schizophrenia (Bauer and Teixeira, 2019, Gilhus and Deuschl, 2019), but the evidence is somewhat ambiguous (Fried et al., 2019, Miller and Goldsmith, 2019, Perry, 2018).

Attesting to the enormous importance of detecting neuroinflammation with molecular imaging is the large array of clinically available TSPO PET radiotracers. Despite this, much is still unknown and controversial about the interpretation of TSPO imaging (Guilarte, 2019), partly due to a lack of sufficient knowledge about its cellular functions and distribution. To date, TSPO has been implicated in a variety of cellular & mitochondrial functions, including biosynthesis of steroids and haeme, porphyrin transport, apoptosis, cell proliferation, and neuromodulation (Papadopoulos et al., 2006, Rupprecht et al., 2010), although some of those have recently been challenged based on the lack of confirmatory findings in studies using TSPO knockout animals (Banati et al., 2014, Middleton et al., 2015, Tu et al., 2014, Wang et al., 2016). In light of all available evidence, there is now an emerging consensus that the primary role of TSPO may be for the regulation of cellular energy turnover processes (Betlazar et al., 2020). As mounting a proinflammatory response by microglia is energetically expensive, this could explain the observed increase in TSPO binding observed in pathological conditions (O’Neill et al., 2016). Additionally, in an inflammatory state, TSPO-rich immune cells from outside the brain (Zhao et al., 2020), such as monocytes and macrophages may infiltrate the brain, and some of the observed central TSPO signal may therefore originate from this peripheral population. The role of TSPO in energy maintenance could also explain its abundance in various other brain cells, all of which have not only immunomodulatory but also energetically demanding roles in the brain, such as ependyma and choroid plexus, which are involved with regulating the cerebrospinal fluid (Hofman and Chen, 2016, Wilson, 1980); the endothelium, which is involved with maintaining the blood brain barrier (Oldendorf et al., 1977); the astrocytes which proliferate and reconfigure during inflammation (Liddelow and Barres, 2017); and even neurons whose increased activation was recently reported to increase TSPO (Notter et al., 2020). All of this greatly complicates the interpretation of ‘neuroinflammation’ based solely on TSPO image data.

A single systemic administration of low-to-moderate dose of the bacterial endotoxin, lipopolysaccharide (LPS), is known to induce transient microglia proinflammatory activation (Biesmans et al., 2013, Buttini et al., 1996), as well as the characteristic “sickness behaviour”, in a variety of experimental animals and humans (Dantzer et al., 2008, DellaGioia and Hannestad, 2010, Ferreira Mello et al., 2013, Park et al., 2011, Schedlowski et al., 2014). This acute LPS challenge approach has been utilized to study neuroinflammation, as measured by an increase in the TSPO PET signal, in humans (Sandiego et al., 2015), primates (Hannestad et al., 2012) and mice (Vignal et al., 2018). As none of those studies were able to explore cellular origins of the signal changes, they mostly attributed them to be of microglial origin. Moreover, many human PET studies continue to view TSPO signal as exclusively labelling changes in microglial activation. However, altered TSPO expression should be understood as the total result of the interactions between various cell types involved in neuroimmune modulation, in addition to microglia (Lee et al., 2020).

To address this point, we aimed to more comprehensively examine the cellular contributors of the TSPO signal after an acute peripheral LPS challenge in rats. We aimed to confirm the upregulation of TSPO utilising *in vivo* PET imaging, followed by quantitative *ex vivo* autoradiographic, immunohistochemical and *in situ* hybridization examination of the CNS tissue, in order to map the contribution of glial, neuronal, endothelial and peripheral monocyte-derived macrophage cells attributing to the overall PET signal.

## 2 Methods

### 2.1 Animals

Male Sprague-Dawley (SD) adult rats weighing 250–300 g (Charles River, UK) were used in all experiments. Rats were housed under controlled environmental conditions (22 ± 1 °C and a 12-h light/12-h dark cycle) with free access to food and water. This study was ethically reviewed and conducted in accordance with Animals (Scientific Procedures) Act 1986 and the GSK Policy on the Care, Welfare and Treatment of Animals.

#### 2.1.1 Intracerebral LPS (ic LPS)

We initially used a rat model of intracerebral administration of LPS (ic LPS model) since it has been extensively used for the evaluation of brain nuclear medicine imaging tracers (Dickens et al., 2014, Ory et al., 2016, Sridharan et al., 2017, Venneti et al., 2007) and is known to induce a robust focal inflammatory lesion (Sridharan et al., 2017). Rats (n = 11) were treated with unilateral stereotaxic injection of 1 µg (in 0.5 µg/µL final concentration) of LPS from Escherichia coli 0111: B4 (Sigma) into the right dorsal striatum (coordinates: +3 mm ML, −4 mm DV, +1 mm AP to the Bregma) using a 2 μL Neuros™ microsyringe (Hamilton, USA) and injection rate of approx. 0.5 μL/min (Espinosa-Oliva et al., 2013). PET image acquisition was carried out 4 days after intracerebral injection of LPS using [^11^C]PK11195 (n = 3) and [^18^F]DPA-714 (n = 4) after which the brains were removed for autoradiography analysis. For corroborative immunofluorescence a different cohort of rats (n = 4) were utilised. See also experimental design in Table 1.

**Table 1:** Details of experimental procedures and number of animals (n) used to generate data shown in this report. LPS: lipopolysaccharide; PFA: paraformaldehyde.

Model	PET Scan/Tracer	Brain processing method	Post-mortem method
ic LPS (11) intracranial LPS (1 µg) injection into the right striatum; scanned and/or sacrificed after 4 days	No tracer administered (4)	Perfused with PFA	Immunofluorescence (4)
	[^11^C]PK11195 (3)	Flash frozen	[^3^H]PK11195 autoradiography (3)
	[^18^F]DPA-714 (4)	Flash frozen	[^3^HDPA-714 autoradiography (4)
ip systemic LPS (40) systemic intraperitoneal injection with LPS (0.5 mg/kg) or saline (veh); scanned and/or sacrificed after 1 day	[^18^F]DPA-714 (8 veh, 8 LPS)	Half flash frozen, half post-fixed in PFA	Immunohistochemistry and [^3^H]DPA-714 autoradiography (5–6 veh, 5–7 LPS)
	No tracer administered (12)	Perfused with PFA	Immunofluorescence (6 veh, 6 LPS)
	No tracer administered (12)	Perfused with PFA	RNAscope (5–6 veh, 5–6 LPS)

#### 2.1.2 Systemic intraperitoneal LPS (ip LPS)

In order to induce neuroinflammation via a peripheral administration of LPS, rats (n = 40) were injected intraperitoneally (ip) with either LPS from Escherichia coli 0111: B4 (Sigma) (0.5 mg/kg, ip), or vehicle injection (saline 1 mL/kg) as a control. PET imaging was carried out 24 hours following challenge using [^18^F]DPA-714 (n = 8/group) and the brains were removed for immunohistochemistry and autoradiography analyses. For immunofluorescence and RNAscope *in situ* hybridization, two different cohort of rats (n = 6/group and n = 5–6/group respectively) were utilised. See experimental design in Table 1. The timing of the PET imaging  (24 hours after peripheral LPS administration), was chosen as this represents a period where the occurrence of depressive-like behavior has previously been observed (Dantzer et al., 2008, Park et al., 2011).

### 2.2 Radiochemistry

#### 2.2.1 [^11^C]PK11195 synthesis

[^11^C](R)-PK11195 was produced at the PET Centre, St Thomas’s Hospital, London, UK. Carbon-11 was used to radiolabel PK11195 with an overall synthesis time of 35 min from the end of bombardment. (R)-N-Desmethyl PK11195 was the precursor used for the synthesis.  The process involved (a) synthesis of [^11^C]CH_3_I from [^11^C]CO_2_ using TracerLab FX MeI, (b) transfer of [^11^C]CH_3_I to Trasis AiO module, (c) reaction of [^11^C]CH_3_I with KOH (4 mg) and precursor (1 mg) in DMSO at room temperature for 2 min, followed by (d) semi-preparative HPLC purification on Gemini C18 250x10mm 10µ, Phenomenex and finally (e) product formulation on Sep-Pak C18 light and sterilisation on Sterifix Paed filter. On average, 0.5–1 GBq [^11^C]PK11195 was obtained from 20 GBq of [^11^C]CO_2_ (greater than 95% radiochemical purity and greater than 50 GBq/µmol) with activity yields of 4 ± 2%.

#### 2.2.2 [^18^F]DPA-714 synthesis

[^18^F]DPA-714 was synthesized at the Centre for Radiopharmaceutical Chemistry, University College London, UK, using a Trasis AllinOne synthesizer. [^18^F]fluoride in water was trapped on a Sep-Pak® QMA cartridge and subsequently released with 0.8 mL of a solution of a) Kryptofix 222 (27 mM) in acetonitrile (650 µL) and b) potassium carbonate (9.8 mM) in water (150 µL). Following azeotropic drying, the precursor solution containing toluene-4-sulfonic acid 2-[4-(3-diethylcarbamoylmethyl-5,6-dimethyl-pyrazolo-[1,5-a]pyrimidin-2-yl)-phenoxy]-ethyl ester) (3 mg) in DMSO (900 µL), was added. The resulting solution was heated at 120 °C for seven minutes. After cooling, the reaction was quenched with ammonium acetate solution (0.1 M) and purified by HPLC using an Agilent Zorbax RP18-e column (250 × 9.4 mm, 5 µm) at room temperature. The mobile phase consisted of 45% *w/v* ammonium acetate solution (0.1 M) and 55% *v/v* methanol. The isolated product was trapped on a Sep-Pak® SPE tC18 Plus cartridge, and the radiolabelled product was formulated into an injectable using 10% *v/v* ethanol in saline (0.9%). The product was sterilized by filtration. The radiochemical purity of [^18^F]DPA-714 always exceeded 99%, and the molar activity of the [^18^F]DPA-714 was ~ 1 TBq/µmol.

### 2.3 PET image acquisition

Image acquisition was conducted on both rat models of neuroinflammation (i.e. ic LPS and ip LPS). Anaesthesia was induced with 4% isoflurane and maintained at 2–2.5% of isoflurane in 100% O_2_.

#### 2.3.1 Ic LPS rat image acquisition

Four days after ic LPS, rats were placed in a prone position and scans conducted utilising a nanoScan microPET-CTPlus (Mediso, Hungary) scanner following intravenous administration of [^18^F]DPA-714 (12.03 ± 0.81 MBq) or [^11^C]PK11195 (11.68 ± 1.04 MBq). Dynamic PET images were acquired over 60 min for [^18^F]DPA-714 and 40 min for [^11^C]PK11195. Scans were obtained at 400–600 keV energy window, 5 ns coincidence time and coincidence mode of 1–5. A CT scan was performed at standard frame resolution (512 × 512 pixels), 55 kVP tube voltage, 600 ms of exposure time and 360° projections. Reconstruction was carried out using ordered subset expectation maximization (OSEM) iterative reconstruction algorithm (propriety software, Mediso Ltd.); binning intervals 1 x 60 s; 1 x 240 s; 5 x 300 s, 1 x 600 s for [^11^C]PK11195 and 1 × 60 s; 1 × 240 s; 7 × 300 s, 2 × 600 s for [^18^F]DPA-714. Corrections for decay, randoms, crystal dead time, detector normalisation and attenuation correction were implemented. Images were reconstructed with a voxel size of 0.25 × 0.25 × 0.25 mm^3^ for CT and 0.4 × 0.4 × 0.4 mm^3^ for PET. We compared ipsilateral (lesioned) side of the brain with the contralateral (non-lesioned) side as a control, since contralateral side shows no inflammatory reaction (activation of microglia or astrocytes) 4 days after ic LPS (Supplemental Figure 1) .

Ipsilateral/contralateral ratios were calculated by dividing the area under the curve (AUC) for basal ganglia in the ipsilateral vs. in the contralateral over the entire time frame.

#### 2.3.2 Ip LPS rat image acquisition

On the day of image acquisition, twenty-four hours (±2 h) following systemic administration of LPS or vehicle, rats were surgically (under 2.5% isoflurane anaesthesia) prepared by implanting a femoral venous and arterial catheter for radiotracer administration and blood sampling, respectively. They were then placed in a supine position and scanned for 60 mins following intravenous administration of [^18^F]DPA-714 (12.79 ± 1.75 MBq). Image acquisition and reconstruction was carried out as described in the previous section for [^18^F]DPA-714 PET. Arterial blood samples (20 μL per sample) were collected and plasma isolated by centrifugation at 3,000 rpm, room temperature for 5 min. Radioactivity in whole blood and plasma samples were measured by gamma counting, decay corrected, and plotted using Graph-Pad Prism 8 (San Diego, CA, USA) to calculate AUC. The plasma and whole blood radioactive counts obtained were converted to MBq using a calibrated conversion scale, and % injected dose in MBq/g (%ID/g) calculated for each rat.

### 2.4 PET image analyses

Images were analysed using VivoQuant 2.0 (Invicro LLC) software. The 3D rat brain atlas template was used to extract the radiotracer time-activity curves (TACs) in 13 regions of interest (ROI; olfactory, cortex, basal ganglia, corpus callosum, hippocampus, thalamus, amygdala, hypothalamus, midbrain, septal area, ventricles, white matter and cerebellum) and also as a whole brain ROI. Standardized uptake values (SUV) were calculated by dividing the image derived concentration (in kBq/cc) with the ratio of the injected dose in MBq to the body weight (in g).

### 2.5 Tissue collection

Immediately following scanning, ip LPS rats were euthanized by decapitation, whole brains excised and divided into two hemispheres. One hemisphere was flash frozen in chilled isopentane (−35 °C) and stored at − 80 °C until required for autoradiography. The other hemisphere was placed in 4% *w/v* paraformaldehyde (PFA) for 24 h, followed by immersion in 30% *w/v* sucrose solution and stored at 4 °C until required for assessment by immunohistochemistry. For the ic LPS rats, immediately following scanning rats, whole brains were flash frozen in chilled isopentane (−35 °C) and stored at −80 °C until required for autoradiography (Table 1).

### 2.6 Autoradiography

Brain hemispheres from the ic LPS (n = 4), ip LPS (n = 5–6) and ip vehicle (n = 5–7) rats were coronally cryosectioned at 20-µm thickness, mounted directly onto glass slides and processed for autoradiography as described previously (Mancuso et al., 2019). Autoradiographical assessment of the ic LPS was conducted utilising both [^3^H]PK11195 and the second-generation TSPO radiotracer [^3^H]DPA-714, whilst we only used [^3^H]DPA-714 for the ip LPS model.

Briefly, slides were incubated with 100 mM Tris-HCl, containing 1 nM [^3^H]PK11195 (specific activity 82.7 Ci/mmol, Perkin Elmer, #NET885001MC) for 30 min, or 1 nM [^3^H]DPA-714 (specific activity 40.8 Ci/mmol, custom tritiated by RC TRITEC, Switzerland) for 60 min, washed twice for 6 min in 100 mM Tris-HCl, rinse-dipped in dH_2_O and air dried. Non-specific binding (NSB) for both radiotracers were determined on adjacent sections in the presence of unlabelled PK11195 (20 µM; Sigma-Aldrich, #85532–75-8). Slides were exposed to tritium-sensitive film (Amersham Hyperfilm MP, GE Healthcare) in autoradiography cassettes together with a set of tritium standards ([^3^H] Microscale, American Radiolabelled Chemicals, #art-0123A) for 6 weeks ([^3^H]PK11195) or 4 weeks ([^3^H]DPA-714). Quantitative analysis was performed using MCID image analyzer (Image Research, Canada), and the brain structures were identified using the rat brain atlas of Franklin and Paxinos (1997). All regions of interest (ROI) were analyzed by freehand drawing tools in two consecutive sections per brain (i.e. adjacent total and NSB sections). Certain animals were excluded from the analysis given the difficulty to sample the ROI.

### 2.7 Histology

Immunohistochemistry for TSPO (NP155 antibody) was performed in the remaining hemispheres from ip LPS (n = 8) and vehicle (n = 8) rats that underwent [^18^F]DPA-714 PET study, to determine brain TSPO protein expression (Table 1). NP155 antibody was generously provided by Dr Makoto Higuchi and extensively evaluated by (Ji et al., 2008) and (Owen et al., 2017).

Double immunofluorescence of TSPO (NP155) and Iba1 (a marker for both microglia and macrophages) was conducted on rat brain sections acquired from a non-imaged rat cohort from ip LPS and vehicle (n = 6/group). The animals were transcardially perfused with PBS following 4% *w/v* PFA, 4 days after intracerebral injection of LPS or 24 h after systemic administration of LPS/vehicle. Brains were excised placed in 4% *w/v* PFA for 24 h, followed by immersion in 30% *w/v* sucrose solution and stored at 4 °C.

Brains were cut at 35 µm thickness in HM 430 Sliding Microtome (Thermo Fisher Scientific). Free floating sections were incubated in citrate buffer pH = 8 at 80 °C for 30 min and 0.3% *v/v* H_2_O_2_ to block for endogenous peroxidase activity (only for bright field immunohistochemistry), and with 10% *w/v* milk and 0.3% *v/v* Triton X‐100 in TBS for 40 min. After rinses with tris buffered saline (TBS), sections were incubated overnight at 4 °C with rabbit anti‐TSPO antibody (1:2000 in blocking solution, NP155) for bright field immunohistochemistry and with a mix of primary antibodies for immunofluorescence (see Supplemental Table 1). After washes with TBS, sections were incubated with the appropriate biotinylated (Vector Labs) or Alexa-conjugated secondary antibodies (see Supplemental Table 1).

#### 2.7.1 Immunohistochemistry

For bright field immunohistochemistry, following rinsing, the sections were incubated with Vectastain Elite ABC peroxidase kit (Vectors Labs) and visualized with diaminobenzidine (DAB) precipitation. Sections were mounted with DPX and images were captured at 40x magnification with photomicrographs captured using a Virtual Slide Microscope VS120 (Olympus Life Science). ROIs from the olfactory area, hippocampus, substantia nigra, periaqueductal grey midbrain and the cerebellum (3–4 sections/ROI) were analysed by measuring the percentage area covered by TSPO in 1700 × 1700 pixels images using ImageJ (NIH, USA). Certain animals were excluded from the analysis given the difficulty to sample the ROI.

#### 2.7.2 Immunofluorescence

For immunofluorescence, sections were counterstained with 4′,6-diamidino-2-phenylindole (DAPI) and mounted with FluorSave™ mounting medium (Calbiochem, #345789–20). Imaging was performed using an inverted spinning disc confocal microscope system (Nikon Eclipse T1). For relative quantification of immunofluorescence, one 4 × 4 large photomicrograph containing series of ~ 10 µm deep Z stacks corresponding to ~ 12 optical sections at 60x fields were capture from one dorsal CA1 of the hippocampus section (Bregma −3.24 mm) and one substantia nigra section (Bregma −3.24 mm) per animal. For each image, the total number of (i) Iba1+ cells (microglia/macrophages), (ii) TSPO+ cells (all cells expressing the TSPO protein), (iii) TSPO+Iba1+ cells (microglial/macrophages cells expressing TSPO protein), and (iv) TSPO+Iba1- cells (all other cells, excluding microglial/macrophages cells expressing TSPO protein), were counted using NIS Elements Nikon software by thresholding using DAPI staining nuclei as a counterstaining.

To measure the TSPO expression of other cell types (Iba1- cells), such as astrocytes, neurons or endothelial cells, to the overall LPS induced TSPO expression, double immunofluorescence of TSPO (NP155) and GFAP (astrocytes) and triple immunofluorescence of TSPO (NP155), NeuN (neurons) and CD31 (vascular endothelial cells) were performed, following the protocol previously described (see Supplemental Table 1 for antibodies used).

### 2.8 RNAscope *in situ* hybridization

Finally to further investigate *Tspo* gene expression in different immune-cell types, such as microglia and infiltrated monocytes-derived macrophages, and to overcome the limitations in the use of reliable histological markers to discriminate these two immune cells populations, RNAscope Multiplex Fluorescence V2 technology for *in situ* hybridization was used to simultaneously detect three RNA targets (*Tspo*, *Tmem119* and C*cr*2) on fresh-frozen brain sections from vehicle and LPS-treated rats. RNAscope *in situ* hybridization was performed according to the manufacturers protocol for RNAscope Multiplex Fluorescent Reagent Kit v2 (Advanced Cell Diagnostics, #320293), using 3 different probes against the genes of interest (see Supplemental Table 2). For quantification, images were acquired as previously described from one dorsal CA1 of the hippocampus section (Bregma −3.24 mm) and one substantia nigra section (Bregma −3.24 mm) per animal. Analysis was performed using HALO Image Analysis Software (Indica Lab) to quantify the number of *Tspo* mRNA copies following a cell-type discrimination based on the expression of a recently discovered specific microglial marker in the brain; *Tmem119* that is reported to differentiate activated microglia from monocyte-derived macrophages (Bennett et al., 2016); and C*cr*2, a marker that although expressed in both microglia and monocyte-derived macrophages, is primarily expressed in monocyte-derived macrophages (Haage et al., 2019, Li et al., 2019). *Tmem119* mRNA expression is highly enriched in the CNS and is abundandly expressed in microglial cell population (Bennett et al., 2016, Van Hove et al., 2019). However, *Tmem119* is co-expressed in a very low proportion in monocyte-derived macrophages (Li et al., 2019, Van Hove et al., 2019). In order to ensure specific selection of microglia and monocyte-derived macrophages and to minimise nonspecific hybridization events, we defined a specific threshold to distinguish these two cell populations: microglial cells were defined as *Tmem119* positive (more than 5 *Tmem119* mRNA copies) and *Ccr2* negative (less than 5 *Ccr2* mRNA copies); and monocyte-derived macrophages as *Tmem119* negative (less than 5 *Tmem119* mRNA copies) and *Ccr2* positive (more than 5 *Ccr2* mRNA copies) (Fig. 5d).

## 3 Statistics

Time activity curve - standardised uptake value (TAC SUV) data from both the ic LPS and ip LPS rat models, and AUC from the TACs of [^18^F]DPA-714 in the ip LPS model, were analysed using a mixed-effects model ANOVA with ROI or time as within-subject factor and treatment as between-subject factor followed by *post hoc* Sidak tests.

For the ic LPS model, AUC measured in contralateral and ipsilateral striatum were confirmed to follow a normal distribution with Shapiro-Wilk normality tests and were subsequently analysed using paired t-tests and ipsilateral/contralateral ratios with an unpaired *t*-test.

The remaining data were also analysed by mixed-effects ANOVA with cells or ROI as within-subject factor and treatment as between-subject factor followed by *post hoc* Sidak tests. Number of Iba1+ cells and TSPO+ cells were analysed using unpaired *t*-test. Quantitative data are expressed as mean ± SEM. All statistical analyses were carried out using GraphPad Prism (version 8), and p value < 0.05 was considered to be statistically significant.

## 4 Results

### 4.1 Increased ipsilateral striatal uptake of both [^11^C]PK11195 and [^18^F]DPA-714 with ic LPS treatment

A robust focal inflammatory response following ic LPS has been detected in the present study (Supplemental Figures 1 and 2) including increased activation of microglia and astrocytes, as well as upregulation of TSPO, accompanied by induction of mRNA expression for various proinflammatory cytokines such as *Tnf-α*, *Il-6*, *Il-1β*, intercellular adhesion molecules (*Icam-1*) and peripheral monocyte recruitment (*Ly6c*) (see Supplemental Figure 2). This response was expected and aligns with previous reports studying different TSPO radiotracers (Ory et al., 2015, Sridharan et al., 2017). We aimed to replicate these findings with two TSPO radiotracers, [^11^C]PK11195 and [^18^F]DPA-714, in order to select the more sensitive radiotracer for the peripherally-induced neuroinflammation study (ip LPS), which was anticipated to present a more physiological neuroinflammatory phenotype.

**Fig. 1:**
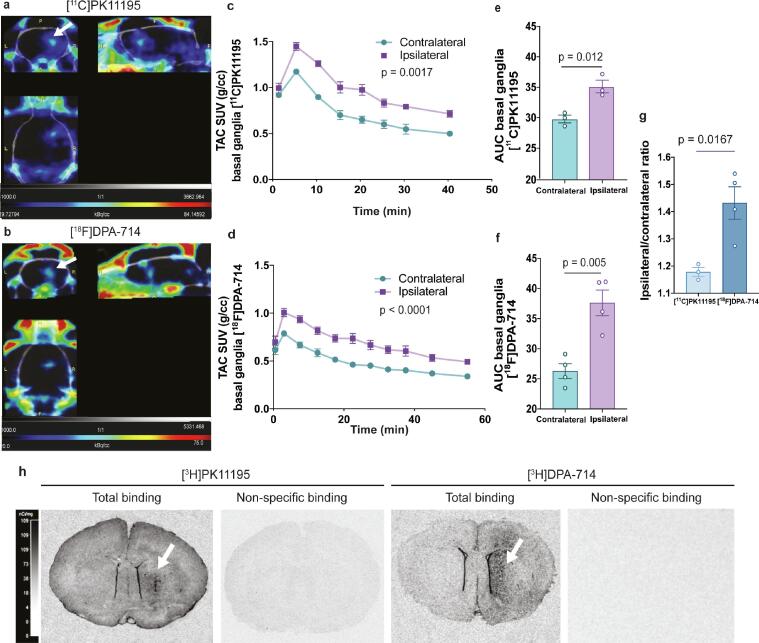
Comparison of [^11^C/^3^H]PK1195 and [^18^F/^3^H]DPA-714 uptake in the ic LPS treated rat. Representative images of (a) [^11^C]PK11195 and (b) [^18^F]DPA-714 showing radiotracer uptake into the lesioned area of the brain, as indicated with white arrows. Colorimetric scales indicate red as maximal and blue as minimal SUV. TAC SUV for the lesioned basal ganglia region are shown on the y-axis over time for (c) [^11^C]PK11195 and (d) [^18^F]DPA-714, for ipsilateral and contralateral basal ganglia ROI in rats unilaterally lesioned with LPS. Area under the curve (AUC) from the TACs of (e) [^11^C]PK11195 and (f) [^18^F]DPA-714 in the basal ganglia are shown in the y-axis; along with (g) the ipsilateral/contralateral ratios calculated over the entire time frame. (h) Representative *ex vivo* autoradiographs of [^3^H]PK11195 and [^3^H]DPA-714 bound to the rat CNS along with the respective non-specific binding, 4 days after intracerebral administration of LPS. LPS-induced lesion can visualised in these sections (arrows). Results are expressed as mean ± SEM. SUV: standard uptake value; TAC: time activity curve.

We compared ipsilateral (lesioned) side of the brain with the contralateral (non-lesioned) side as a control, since contralateral side shows no inflammatory reaction (activation of microglia or astrocytes) 4 days after ic LPS (Supplemental Figure 1). A significant increase in uptake of radiotracer was observed in the LPS-lesioned hemisphere compared to the non-lesioned hemisphere for both [^11^C]PK11195 and [^18^F]DPA-714 (F (1, 4) = 55.27 , p = 0.0017 and F (10, 60) = 122.3, p < 0.0001, respectively). Additionally, a significant effect of interaction time × hemisphere (F (7, 28) = 2.855, p = 0.0222; F (10, 60) = 4.148, p = 0.000224) was observed in the TACs for both [^11^C]PK11195 and [^18^F]DPA-714 in the basal ganglia (Fig. 1a-d). As expected, ic LPS induced a significant increase in both [^11^C]PK11195 and [^18^F]DPA-714 uptake in the basal ganglia region of interest, where the AUC from the TACs confirmed a significantly higher uptake of both [^11^C]PK11195 and [^18^F]DPA-714 in the LPS-lesioned striatum vs. the non-lesioned striatum, 4 days after ic LPS (t (2) = 9.558, p = 0.012; t (3) = 7.456, p = 0.005, Fig. 1e,f). The ipsilateral/contralateral ratio was significantly higher in case of [^18^F]DPA-714, demonstrating better signal, compared to [^11^C]PK11195 (t (5) = 3.533, p = 0.0167, Fig. 1g). The superiority of DPA-714 was confirmed by *ex vivo* autoradiography using [^3^H]PK11195 and [^3^H]DPA-714, where both showed increased binding in the LPS-lesioned side, but [^3^H]DPA-714 signal is demonstrably clearer (Fig. 1h). Subsequently, [^18^F]DPA-714 was selected to assess neuroinflammation in the systemic LPS rat model. No significant differences in [^18^F]DPA-714 SUV AUC values were observed in any other ROI assessed in the LPS-lesioned hemisphere compared to the non-lesioned hemisphere, although there were a few regions that demonstrated elevated uptake in the lesioned-hemisphere, such as the cortex, corpus callosum, thalamus, amygdala, ventricles and white matter (Supplemental Table 3). The effect of the injection procedure at this particular time-point was assessed by immunofluorescence analysis of Iba1 and TSPO expression in ic vehicle- injected rats. No significant differences were detected in the ipsilateral vs. contralateral side of the brain in Iba1 and TSPO expression 4 days after intrastriatal vehicle injection (see Supplemental Figure 1c).

### 4.2 Increased global uptake of [^18^F]DPA-714 with ip LPS treatment

**Fig. 2:**
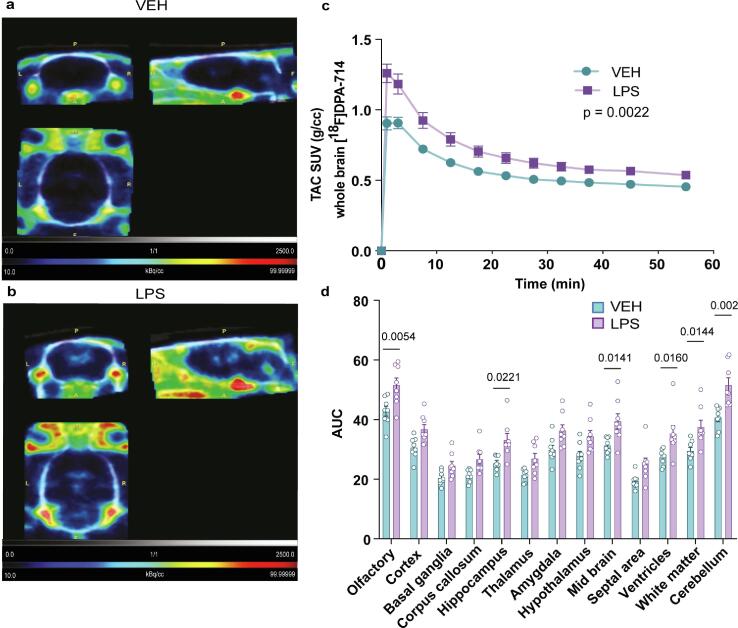
Increased [^18^F]DPA-714 uptake induced by ip LPS administration. Representative images of [^18^F]DPA-714 uptake (a) in the ip vehicle or (b) LPS-treated rats. Colorimetric scales indicate red as maximal SUV and blue as minimal. (c) SUV TACs of [^18^F]DPA-714 uptake for vehicle- and LPS- treated rats as a “whole brain” ROI. (d) Area under the curve (AUC) values generated from the TACs of specified ROIs. Results are expressed as mean ± SEM. SUV: standard uptake value; TAC: time activity curve.

*In vivo* microPET TAC data from ip LPS rats showed a significantly increased overall whole brain uptake of [^18^F]DPA-714 compared to vehicle treated rats (Fig. 2a-c), a significant effect of treatment (F (1, 14) = 14.02, p = 0.0022), time (F (2.306, 32.28) = 310.7, p < 0.0001) and interaction treatment × time (F (11, 154) = 8.827, p < 0.0001) was found. In the AUC of [^18^F]DPA-714 SUV, we note a significant effect of treatment (F (1, 14) = 10.46, p = 0.0060), ROI (F (12, 168) = 198.77 , p < 0.0001), and interaction treatment × ROI (F (12, 168) = 2.47, p = 0.0053). Upon *post hoc* testing we identified the biggest increases in LPS- vs. vehicle-treated rats in the olfactory (20.4%, p = 0.0054), hippocampal (30.4%, p = 0.0221), mid brain (25.7%, p = 0.0141), ventricle (29.2%, p = 0.0160), white matter (27.3%, p = 0.0144) and cerebellar (26.8%, p = 0.002) ROIs (Fig. 2d).

Importantly, there was no significant difference between the LPS- and vehicle-treated rats in either the peripheral distribution of the radiotracer in the blood or plasma (Supplemental Figure 3a); or in the injected dose/weight (ID/g) of the radiotracer (Supplemental Figure 3b).

### 4.3 Increased global binding of [^3^H]DPA-714 to CNS structures following ip LPS treatment

**Fig. 3:**
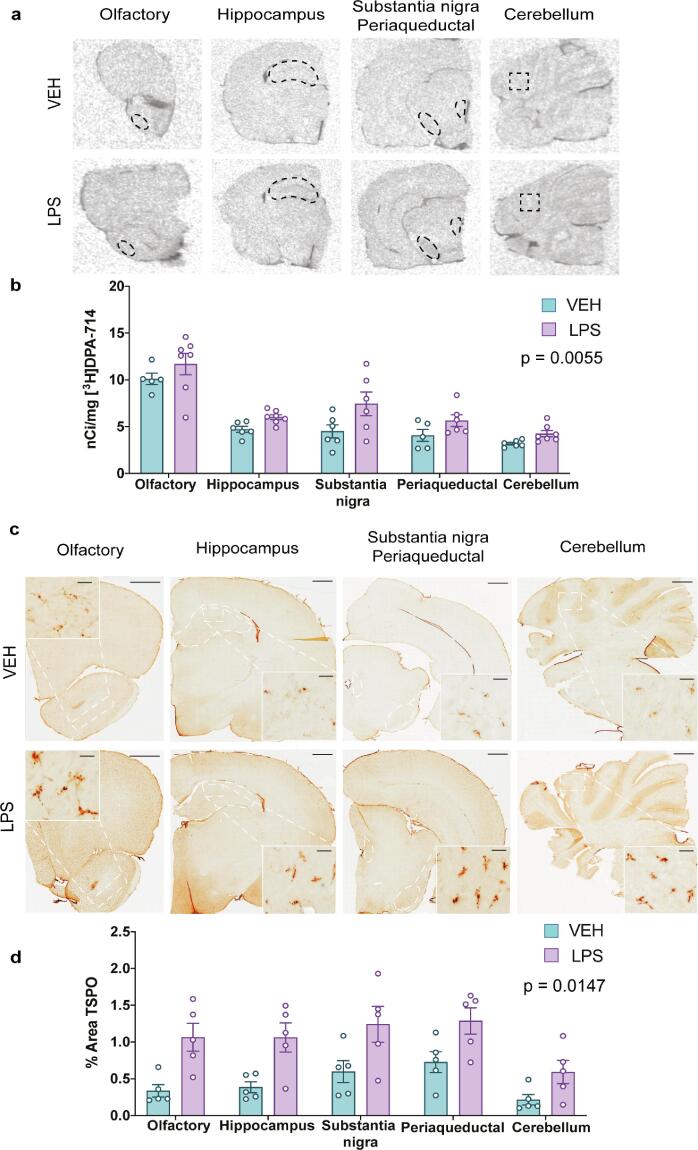
[^3^H]DPA-714 *ex vivo* autoradiography and TSPO immunohistochemistry in ip LPS- and vehicle-treated rats. (a) Representative autoradiographs of [^3^H]DPA-714 in the vehicle- and ip LPS- treated rats with coronal brain sections showing the placement of selected ROIs that were analyzed. (b) Level of bound [^3^H]DPA-714 (nCi/mg) in vehicle- and LPS-treated rats (n = 5–7/group). (c) Representative images of TSPO immunostained coronal sections of the vehicle- and LPS-treated rats. Scale bar = 1 mm magnified inset = 20 μm. (d) Quantification of percentage area TSPO immunostained for the same ROIs (n = 5–7/group). Results are expressed as mean ± SEM.

To corroborate the results obtained by [^18^F]DPA-714 PET in ip LPS rats, we performed [^3^H]DPA-714 *ex vivo* autoradiography on the brain tissue from the imaged animals. Densitometry analysis confirmed a significant 32% overall increase in [^3^H]DPA-714 binding in the whole brain (all ROIs, 7.01 ± 0.73 nCi/mg LPS, 5.31 ± 0.47 nCi/mg VEH). A significant effect of treatment (F (1, 11) = 11.84, p = 0.0055) and ROI was observed (F (4, 40) = 31.69, p < 0.0001), but no significant treatment × ROI interaction was found (F (4, 40) = 0.56, p = 0.6885). Collectively, [^3^H]DPA-714 uptake was found to be increased in all ROIs assessed in the LPS treated-rats compared to vehicle-treated controls, with a qualitative observation suggesting this to be the greatest in the substantia nigra (65.5% increase compared to vehicle-treated rats) and lowest in the olfactory area (15.6%, Fig. 3a-b).

### 4.4 Increased regional brain TSPO expression with ip LPS treatment

Brain tissue from the [^18^F]DPA-714 PET experiment was further investigated for TSPO protein expression by immunohistochemistry. To this end, olfactory, hippocampus, midbrain (including substantia nigra and periaqueductal grey) and cerebellum-containing hemibrains sections from LPS- and vehicle-treated PET-imaged rats were immunostained with an anti-TSPO antibody (Supplemental Table 1). Using thresholding, we detected a significant effect of treatment (F (1, 8) = 9.60, p = 0.0147) and ROI (F (4, 32) = 13.23, p < 0.0001) but no treatment × ROI interaction (F (4, 32) = 1.153, p = 0.3498) on the percent area stained for TSPO, confirming increased TSPO protein expression in LPS-treated rats compared to vehicle- across all ROIs (Fig. 3c,d).

### 4.5 Microglia/macrophages together with astrocytes are the major contributors to increased TSPO expression following ip LPS treatment in rats

To investigate the extent of expression of TSPO within the different types of cells we performed co-localization immunofluorescence. We examined two ROIs, hippocampus and substantia nigra. We selected these ROIs for the following reasons: (1) both were found to have a large increase in TSPO following ip LPS treatment, as measured by PET (Fig. 2) and autoradiography (Fig. 3); (2) previous clinical PET studies have shown significant TSPO changes in these two brain areas of patients with neurodegenerative disease and psychiatric disorders (Haarman et al., 2014, Kreisl et al., 2016, Ouchi et al., 2005) and (3) both brain regions are known to have a particularly high microglia density (De Biase et al., 2017, Tan et al., 2020).

**Fig. 4:**
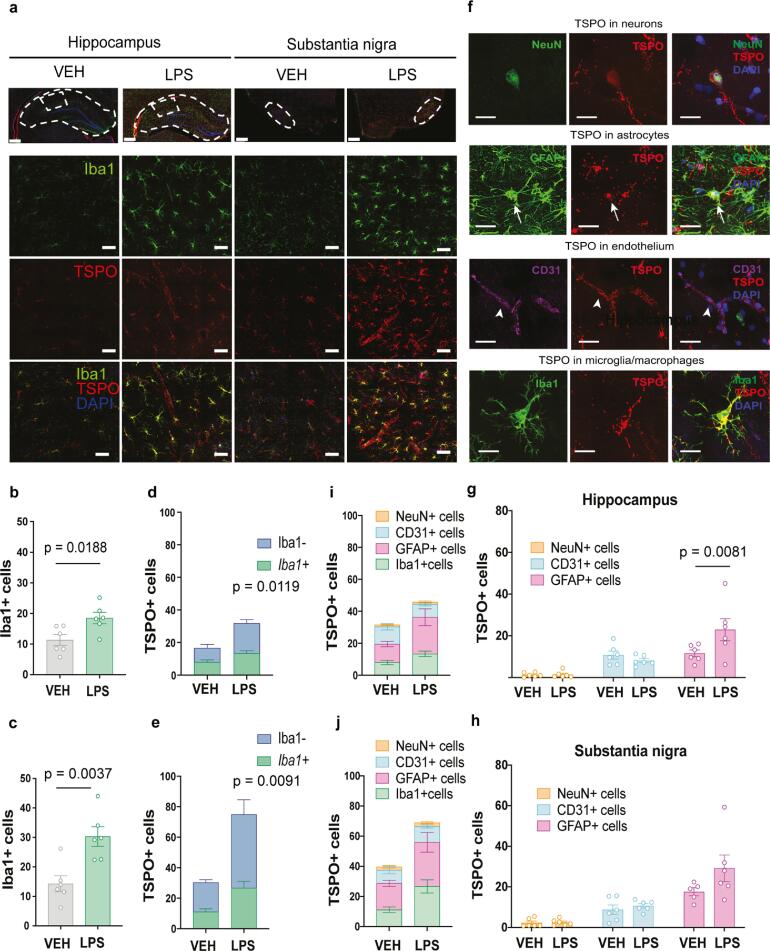
TSPO co-localization with different cell types in ip LPS- or vehicle- injected rats (a) Confocal photomicrographs from Iba1 (green) and TSPO (red)-immunostained hippocampus and substantia nigra sections of ip LPS- and vehicle-treated rats (top left panel). Scale bar whole brain section = 1 mm, scale bar magnification = 50 μm. Nuclear counterstaining was performed using DAPI (blue). Cell counts expressed as Iba1+ cells from (b) the hippocampus and (c) from the substantia nigra, TSPO+Iba+ & TSPO+Iba- cells from (d) the hippocampus and (e) the substantia nigra region from both the ip LPS- and vehicle-treated rats (n = 6/group). (f) Confocal photomicrographs from immunostained sections of ip LPS treated rats: neurons, NeuN (green), astrocytes, GFAP (green), endothelium, CD31 (purple), microglia/macrophages, Iba1 (green) and TSPO (red) (top right panel). Scale bar = 20 μm. Nuclear counterstaining was performed with DAPI (blue). Arrows: cells positive for GFAP and TSPO; arrowheads: cells positive for CD31 and TSPO. Cell counts expressed as TSPO+NeuN+, TSPO+CD31+ & TSPO+GFAP+ cells from (g) the hippocampus and (h) the substantia nigra in the ip LPS- and vehicle-treated rats. Representation of TSPO+Iba1- cells (TSPO+NeuN+, TSPO+CD31+, TSPO+GFAP+) & TSPO+Iba1+ cells in (i) the hippocampus and (j) the substantia nigra. Results are expressed as mean ± SEM. (For interpretation of the references to colour in this figure legend, the reader is referred to the web version of this article.)

We first investigated the cells of microglia/macrophages lineage which were defined as Iba1 positive (Iba1+). We found Iba1+ cells to be significantly increased after LPS treatment in the hippocampus (63%, t (10) = 2.799, p = 0.0188, Fig. 4b) and in the substantia nigra (112%, t (10) = 3.764, p = 0.0037, Fig. 4c).

We next examined the co-localization, defining two types of cells, based on TSPO and Iba1 expression: TSPO positive that are also positive for Iba1 (TSPO+Iba1+), and TSPO positive that are not positive for Iba1 (TSPO+Iba1-). We looked at the effect of LPS treatment, the type of cells, and their interaction. In the hippocampus, we observed significant effect of LPS treatment (F (1, 10) = 9.409, p = 0.0119) and of cell type (F (1, 10 = 5.097, p = 0.0476) but no interaction (F (1, 10) = 2.858, p = 0.1218), indicating that after LPS both TSPO+Iba1+ and TSPO+Iba1- cells increased evenly (Fig. 4d). Similarly, in the substantia nigra, there was a significant effect of LPS (F (1, 10 = 10.42, p = 0.0091) and of cell type (F (1, 10 = 19.49, p = 0.0013) with no significant interaction (F (1, 10) = 4.032, p = 0.0724), although there appeared to be a trend toward a greater increase in Iba1- cells after LPS (Fig. 4e). This suggests that LPS treatment induced an overall increase in various TSPO expressing cells that are not only of macrophage/microglia lineage. It is also interesting to note that even in the vehicle treated rats there was a similar number of TSPO+Iba1- and TSPO+Iba1+ cells, suggesting that in basal conditions there is as much TSPO in microglia/macrophage cells as in other cell types.

In order to investigate the identity of TSPO+Iba1- cells, we performed a further immunofluorescence study: double immunofluorescence of TSPO and GFAP (astrocyte marker), and triple immunofluorescence of TSPO, NeuN (neuronal marker) and CD31 (vascular endothelium marker). These multi-immunofluorescence investigations confirmed the expression of TSPO in astrocytes, neurons and endothelial cells in both the hippocampus and substantia nigra from both LPS and vehicle-treated rats (Fig. 4g,h). Statistical analysis demonstrated a significant effect of cells (F (2, 20 = 29.54, p < 0.0001) and significant cells × treatment interaction (F (2, 20 = 6.431, p = 0.007) in the hippocampus. *Post hoc* analysis confirmed that only the TSPO+GFAP+ cells increased significantly after LPS treatment (p = 0.0081). This trend of increased TSPO+GFAP+ cells also occurred in the substantia nigra, although there was no significant treatment × cells interaction and hence no *post hoc* comparisons were performed. For the substantia nigra there appeared to be only an overall significant effect of cells (F (2, 19 = 28.22, p < 0.0001). Overall these data suggest that although TSPO is present in all the cell types examined, astrocytes appear to be the main non-microglial contributor to the increased expression of TSPO following ip LPS treatment.

### 4.6 LPS-induced increased *Tspo* mRNA expression is limited to microglia specifically in the hippocampus, and to both microglia and monocyte-derived macrophages in the substantia nigra

**Fig. 5:**
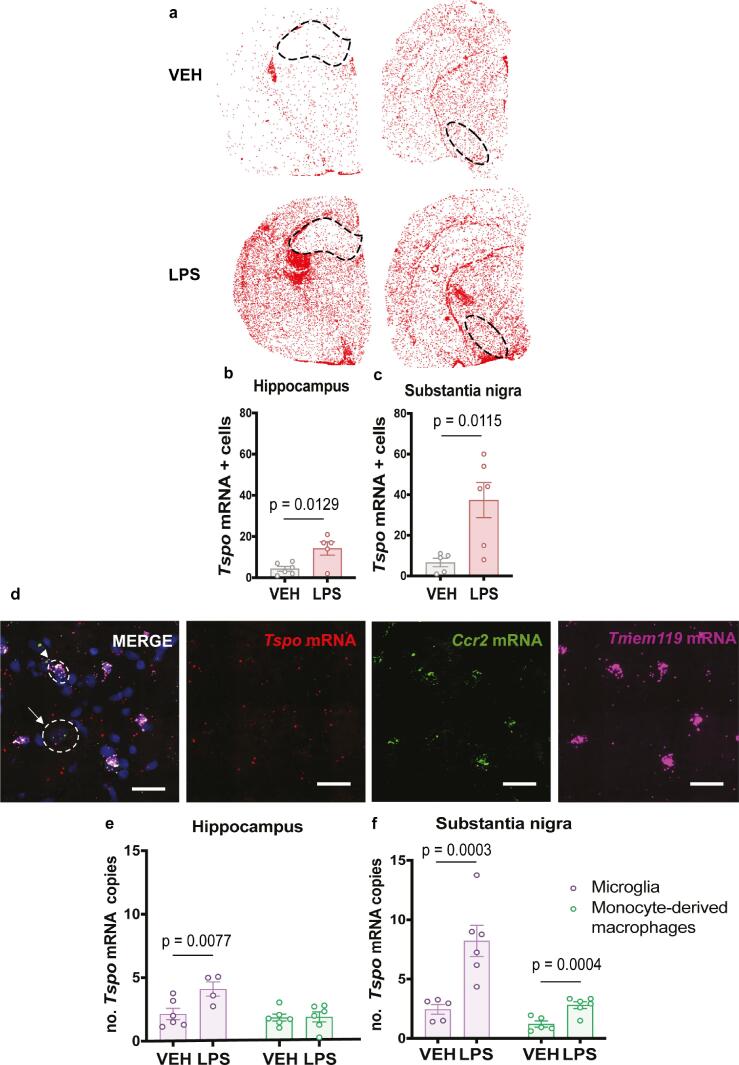
**RNAscope *in situ* hybridization of ip LPS treated rats** (a) Map of *Tspo* hybridization signals in sections at the level of the hippocampus and substantia nigra of LPS- and vehicle-treated rats. Each red dot represents one identified *Tspo* mRNA positive cell. Scale bar magnification = 50 μm. Cell counts expressed as *Tspo* mRNA + cells in (b) the hippocampus and (c) the substantia nigra from ip LPS- and vehicle-treated rats. (d) Confocal photomicrographs from *Tmem119* (purple), *Tspo* (red) and *Ccr2* (green)-hybridize section of ip LPS treated rats. Scale bar = 50 μm. Nuclear counterstaining was performed with DAPI (blue). Arrows: Microglia cells (*Tmem119* positive *Ccr2* negative); arrowheads: monocyte-derived macrophages cells (*Tmem119* negative *Ccr2* positive). Number of *Tspo* mRNA copies in Microglia (*Tmem119* positive *Ccr2* negative) (purple bars) and in monocyte-derived macrophages (*Tmem119* negative*Ccr2* positive) (green bars) from (e) the hippocampus and (f) substantia nigra from ip LPS- and vehicle-treated rats. Results are expressed as mean ± SEM. (For interpretation of the references to colour in this figure legend, the reader is referred to the web version of this article.)

In order to further characterize the identity of TSPO+Iba+ cells in both ROIs and to determine whether they were monocyte-derived macrophages or microglia, RNAscope Multiplex Fluorescence V2 technology for *in situ* hybridization was used to investigate *Tspo* gene expression in these different immune-cell types. Ip LPS treatment caused a significant increase in *Tspo* mRNA positive cells in both the hippocampus and substantia nigra compared to vehicle treated rats (t (9) = 3.091, p = 0.0129; t (9) = 3.164, p = 0.0115, respectively, Fig. 5b,c). For the purposes of distinguishing microglial cells from monocyte-derived macrophages, microglial cells were defined as *Tmem119* positive and *Ccr2* negative; and monocyte-derived macrophages as *Tmem119* negative and *Ccr2* positive (Fig. 5d). In the hippocampus, we observed significant effects of LPS treatment (F (1, 10) = 5.442, p = 0.0418) and of cell type (F (1,9 = 10.08, p = 0.0113) but not of interaction (F (1, 9) = 5.931, p = 0.0376). Similarly, in the substantia nigra, there was a significant effect of LPS treatment (F (1, 9 = 17.66, p = 0.0023) and of cell type (F (1, 9 = 25.79, p = 0.0007), but also of interaction (F (1, 9) = 10.16, p = 0.0110). *Post hoc* analysis of the number of *Tspo* mRNA copies in both cell populations revealed that in the hippocampus, *Tspo* mRNA increased expression was confined to microglia specifically (p = 0.0077, Fig. 5e), but in contrast, the substantia nigra had an elevated number of *Tspo* mRNA copies which were significantly higher in both microglia and monocyte-derived macrophages in LPS-treated rats (p = 0.0003, p = 0.0004, Fig. 5f).

## 5 Discussion

The aim of the current study was to ascertain if *in vivo* PET imaging with the second generation TSPO PET radiotracer, [^18^F]DPA-714, can be used to detect neuroinflammation induced via peripheral admnistration of the endotoxin, LPS, in a rodent model, and also provide insight into the origin of the signal changes in terms of their cellular sources and regional distribution.

We performed an initial study to compare two TSPO PET radiotracers, [^11^C]PK11195 (first generation) and [^18^F]DPA-714 (second generation), and confirmed an increased uptake of both in a focally-lesioned area of the brain. We used a well-characterised model of unilateral intracranial LPS administration to model the focal inflammation, and we performed our measurements 4 days after LPS by when a potentially confounding disruption of the blood brain barrier largely subsides (Sridharan et al., 2017), while microglia are robustly activated around the lesioned area (Supplemental Figure 1) (Choi et al., 2009). These findings corroborated previous studies which demonstrated increased TSPO expression in the same type of model with various TSPO PET radiotracers including [^11^C]PK11195 and [^18^F]DPA-714 (Ory et al., 2016, Sridharan et al., 2017, Ory et al., 2016, Sridharan et al., 2017) and are consistent with published data indicating that [^18^F]DPA-714 appears to be more sensitive than [^11^C]PK11195 to detect TSPO in acute neuroinflammation (Boutin et al., 2013, Chauveau et al., 2009). The increased sensitivity of [^18^F]DPA-714 and other “second generation” TSPO radiotracers over the first generation tracer, [^11^C]PK11195, is becoming increasingly recognised (Alam et al., 2017). Nevertheless the reason some clinical studies continue to use [^11^C]PK11195 may be due to it not suffering from the problematic binding profile, unlike second generation radiotracers wherein approximately third of the population must be excluded due to low binding caused by a particular genetic polymorphism in their human *Tspo* gene (Owen et al., 2012) that does not appear to feature in rodents. Due to the increased sensitivity of the [^18^F]DPA-714 PET tracer demonstrated in this initial study, we selected this PET radiotracer to assess neuroinflammation in the ip LPS rat model.

In the rat model of peripherally induced neuroinflammation, we measured an increased [^18^F]DPA-714 uptake in the whole brain 24 hours after systemic LPS administration. We chose this time-point because of in-house data confirming robust microglial activation and *Tspo mRNA* upregulation (Supplemental Figure 4). In addition, RT-qPCR analysis showed that the cytokine *Tnf-α* and the intercellular adhesion molecule-1 (*Icam-1*) were also elevated in the brain at this time (Supplemental Figure 4), both of which are highly relevant to brain inflammation in context of psychiatric disorders (Capuron and Miller, 2011, Müller, 2019). The increased expression of *Icam-1* particularly points towards activation of endothelial cells as a result of alterations of the blood brain barrier and increased peripheral immune cell recruitment, adhesion and infiltration (Müller, 2019). *Tnf-α* is additionally capable of stimulating microglia to produce monocyte chemoattractant protein-1 (*Mcp-1*) which was also increased in our samples (Supplemental Figure 4) and which is likely to mediate the recruitment of monocytes into the brain (D’Mello et al., 2009). Collectively these data confirm the immune system activation and suggest peripheral immune cell infiltration in the brain parenchyma at this time-point. Interestingly, two earlier PET studies, one in humans (Sandiego et al., 2015) and one in non-human primates (Hannestad et al., 2012) demonstrated increased TSPO binding at the much earlier time-points of 1 and 4 hours following iv LPS administration, respectively. We did not investigate such early time-points because our pilot rat experiments showed no evidence of either microglia hypertrophy or hyperplasia 6 hours after systemic LPS. This is also borne out by the literature where the majority of studies using similar doses of LPS in rodents report activated microglia at 24 hours (Biesmans et al., 2013, Buttini et al., 1996, Hoogland et al., 2015). It is nevertheless possible there are species differences in the timing of microglia and TSPO changes, given that humans are known to be much more sensitive to LPS than rodents (Copeland et al., 2005).

The increased ip LPS-induced binding of DPA-714 to TSPO in the rat brains appeared to be global, in that all ROIs showed a similar increase, of which notably the olfactory, hippocampus, mid brain, ventricles, white matter and cerebellum increased the most. This finding matches the only other published rodent study that reported a similarly widespread global TSPO increase in a mouse after a much higher dose (5 mg/kg ip) of LPS (Vignal et al., 2018). Given that none of the aforementioned studies in any species explored the cellular origins of the TSPO signal in more detail, we followed up the rat PET study by *post mortem* autoradiographic and immunohistochemical examinations.

High resolution autoradiography with [^3^H]DPA-714 corroborated the increased overall TSPO binding in all ROIs following ip LPS treatment (Fig. 3a, b). Immunohistochemical analysis also confirmed increased TSPO expression (Fig. 3c, d), which was particularly evident in the olfactory area, hippocampus and substantia nigra (Fig. 3d). Interestingly, neither TSPO immunohistochemistry nor [^3^H]DPA-714 autoradiography detected any significant TSPO increase in the cerebellum (Fig. 3b, d), whereas there appeared to be a clear cerebellar increase in [^18^F]DPA-714 uptake with *in vivo* PET (p = 0.002, Fig. 2d). This could be due to the complex TSPO distribution in the cerebellum and suggests a possibility that LPS-induced [^18^F]DPA-714 signal is comprised of multiple sources including peripheral TSPO bound to plasma proteins and blood cells (Turkheimer et al., 2015, Veronese et al., 2018), in addition to the brain parenchyma. Despite these potentials confounds, our data demonstrate that peripherally induced neuroinflammation in rats can be detected *in vivo* by [^18^F]DPA-714 PET and corroborated by [^3^H]DPA-714 *ex vivo* autoradiography and TSPO immunohistochemistry.

In order to more precisely explore the cellular specificity of TSPO we performed double immunofluorescence studies combining TSPO with markers of different CNS cells subtypes. We analyzed the hippocampal and the substantia nigra regions since these are the most densely microglia populated areas of the brain (De Biase et al., 2017, Tan et al., 2020) and because several clinical studies have demonstrated changes in TSPO PET signal in these areas of patients with neurodegenerative and psychiatric disorders (Haarman et al., 2014, Kreisl et al., 2016, Ouchi et al., 2005). We first looked at TSPO in microglia/macrophage cells (Iba1 positive cells) versus all other cells (Iba1 negative cells) and found that even in the vehicle treated rats, TSPO expression was approximately evenly distributed between these two classes of cells in both ROIs. Following ip LPS treatment, as expected, there was an overall increase in TSPO+Iba1+ cells, but also a surprisingly large fractional increase (≃1 and 1.5 fold increase in hippocampus and substantia nigra respectively) in the TSPO+Iba1- cells compared to vehicle, confirming the presence of TSPO in the non-microglial cells at rest, and as well as in the inflamed state.

As previous studies have shown a low, albeit positive TSPO expression in non-microglial cells including neurons (Veenman and Gavish, 2000), endothelial cells (Betlazar et al., 2018) and astrocytes (Pannell et al., 2020), in both rodents and humans (Gui et al., 2020, Wimberley et al., 2018), we further examined the contribution of those cells to the TSPO+Iba1- signal. We demonstrated the presence of TSPO in all three cell types, in both LPS- and vehicle treated rats (Fig. 4g, h) corroborating that there appears to be an additional non-microglial expression of TSPO. Of the three cell types (neurons, endothelial cells and astrocytes), TSPO after LPS was only significantly increased in hippocampal astrocytes (p = 0.0081), with a similar trend observed in astrocytes in the substantia nigra (Fig. 4g-j) (p = 0.0497). Neither neurons nor endothelial cells demonstrated an upregulation of TSPO in response to LPS, at least at this time-point, in these two brain regions. These results confirm the findings of several studies describing TSPO upregulation in astrocytes in different neurological disorders (Cosenza-Nashat et al., 2009, Lavisse et al., 2012, Nutma et al., 2019). Our results also confirm our original hypothesis that a significant proportion of TSPO imaging signal arises from cells that are not of microglia/macrophage lineage, even under basal conditions.

One of the limitations of using a cellular marker such as Iba1 to study neuroinflammation is that it does not distinguish between CNS resident microglia and peripherally infiltrating macrophages, due to their common lineage. To further establish the cellular specificity of the TSPO+Iba1+ signal, as microglial cells versus monocyte-derived macrophages, we utilised RNAscope *in situ* hybridization. We found that LPS-induced expression of *Tspo* mRNA in the hippocampus was limited to microglia specifically (1-fold increase), whereas in the substantia nigra *Tspo* mRNA was significantly increased in both microglia (2-fold) and monocyte-derived macrophages (1.3-fold) vs. vehicle (Fig. 5). It is possible that this increased Tspo gene expression in monocyte-derived macrophages induced by LPS in the substantia nigra could be responsible for the more pronounced increase in colocalized TSPO+Iba1+ protein signal from cells in the substantia nigra compared to the hippocampus (Fig. 4). Additional studies to more accurately characterise the regional specificity of Tspo expression in monocyte-derived macrophages are warranted especially in conjunction with blood brain barrier permeability. Another limitation of this study is that we restricted our measurements to a single time-point after LPS. Given the reported differences in timing of neuroinflammatory changes after LPS and similar peripheral inductors, it would be of interest to conduct a study combining longitudinal PET imaging with cellular/molecular characterisation of signal changes to capture the timecourse of the immune response and regional brain effects.

## 6 Conclusions

Overall, our data have shown an increased [^18^F]DPA-714 signal following a peripheral challenge with LPS, with analysis revealing this increased signal to arise from both microglia and non-microglia cellular sources in the specific brain regions that were examined. It is important to note that the LPS paradigm utilised in these studies is a model of peripherally-induced inflammation that subsequently causes a neuroinflammatory state but that cannot be directly related to human neurodegenerative disease or psychiatric disorders. It will therefore be imperative to investigate TSPO expression on human tissue acquired from diseased patients vs. matched controls, and to examine the changes at different time-points, as there may be more pronounced/different neuroinflammatory responses exhibited in chronic human neurodegenerative disease or psychiatric disorders. Subsequently, our data may only reflect the “tip of the iceberg” for comprehending how the non-microglial component of the TSPO PET signal can be understood and therefore interpreted in neuroinflammation.

Consequently, as a result of these findings we suggest that an important contribution of microglia, astrocytes and peripheral infiltrated cells needs to be considered when assessing neuroinflammation with TSPO PET radiotracers. Moreover, the cellular origin of TSPO signal appears to depend on the brain region examined, and we further propose that this will also be dependant on the type and the time-course of the inflammatory response.

## Ethics approval

This study was ethically reviewed and conducted in accordance with Animals (Scientific Procedures) Act 1986 and the GSK Policy on the Care, Welfare and Treatment of Animals.

## Consent for publication

All the authors agree to the publication of this work.

## Availability of data and material

The datasets generated during and/or analyzed during the current study are available from the corresponding author on reasonable request.

## Funding

This study was part funded by GSK and a grant from the Wellcome Trust (Grant number: 104025/Z/14/Z). The Centre for Radiopharmaceutical Chemistry is funded in part by the Department of Health’s NIHR Biomedical Research Centres funding scheme; K. Sander is additionally funded by Mallinckrodt.

## CRediT authorship contribution statement

**Marta Vicente-Rodríguez:** Conceptualization, Methodology, Formal analysis, Investigation, Data curation, Writing - original draft, Visualization. **Nisha Singh:** Conceptualization, Methodology, Formal analysis, Investigation, Data curation, Writing - review & editing. **Federico Turkheimer:** Formal analysis, Writing - review & editing, Funding acquisition. **Alba Peris-Yague:** Formal analysis, Investigation. **Karen Randall:** Formal analysis, Investigation. **Mattia Veronese:** Formal analysis, Software. Camilla Simmons: Investigation, Resources, Software. **Abdul Karim Haji-Dheere:** Resources. **Jayanta Bordoloi:** Resources. **Kerstin Sander:** Resources. **Ramla O. Awais:** Resources. **Erik Årstad:** Resources. **Diana Cash:** Conceptualization, Methodology, Investigation, Writing - review & editing, Supervision, Project administration, Funding acquisition. **Christine A. Parker:** Conceptualization, Methodology, Writing - review & editing, Supervision, Funding acquisition.

## Declaration of Competing Interest

The authors declare that they have no known competing financial interests or personal relationships that could have appeared to influence the work reported in this paper.
