# Supplementary material for: Resolving the cellular specificity of TSPO imaging in a rat model of peripherally-induced neuroinflammation
Source: Brain Behav Immun. 2021 Aug;96:154–67. doi: 10.1016/j.bbi.2021.05.025 (PMC8323128; doi:10.1016/j.bbi.2021.05.025)
Supplement: Supplementary Data 1 [file mmc1.docx]

Resolving the cellular specificity of TSPO imaging in a rat model of peripherally-induced neuroinflammation.

Marta Vicente-Rodríguez^1,4^*****, Nisha Singh^1,6^, Federico Turkheimer^1, 4^, Alba Peris-Yague^1^, Karen Randall^1,4^, Mattia Veronese^1^, Camilla Simmons^1,4^, Abdul Karim Haji-Dheere^3^, Jayanta Bordoloi^6^, Kerstin Sander^5^, Ramla O. Awais^5^, Erik Årstad^5^, NIMA Consortium*, Diana Cash^1,4#^, Christine A. Parker^1,2, 4#^

*1. Department of Neuroimaging, Institute of Psychiatry, Psychology & Neuroscience, King's College London, London, United Kingdom.*

*2. GlaxoSmithKline, Stevenage, London, United Kingdom.*

*3. PET Centre, St Thomas' Hospital, London, United Kingdom.*

*4. The Wellcome Trust Consortium for the Neuroimmunology of Mood Disorders and Alzheimer's Disease (NIMA).*

*5. Centre for Radiopharmaceutical Chemistry, University College London, London WC1E 6BS, United Kingdom.*

*6. School of Biomedical Engineering and Imaging Sciences, King’s College London, London SE1 7EH, United Kingdom.*

*^#^ contributed equally*

** The complete list of NIMA Consortium members is included in supplementary materials as appendix.*

**Contact information:**

***Correspondence should be addressed to Marta Vicente-Rodríguez:** [marta.vicente_rodriguez@kcl.ac.uk](mailto:marta.vicente_rodriguez@kcl.ac.uk)

**APPENDIX: NIMA members during the sample collection and data analysis period for the BIODEP Study**

Brighton & Sussex University Hospitals NHS Trust

Dominika Wlazly

Cambridgeshire & Peterborough NHS Foundation Trust

Amber Dickinson, Andy Foster, Clare Knight

Cardiff University

Claire Leckey, Paul Morgan, Angharad Morgan, Caroline O'Hagan, Samuel Touchard

GSK

Shahid Khan, Phil Murphy, Christine Parker, Jai Patel, Jill Richardson

Janssen

Paul Acton, Nigel Austin, Anindya Bhattacharya, Nick Carruthers, Peter de Boer, Wayne Drevets, John Isaac, Declan Jones, John Kemp, Hartmuth Kolb, Jeff Nye, Gayle Wittenberg

King’s College London

Gareth Barker, Anna Bogdanova, Heidi Byrom, Diana Cash, Annamaria Cattaneo, Daniela Enache, Tony Gee, Caitlin Hastings, Melisa Kose, Giulia Lombardo, Nicole Mariani, Anna McLaughlin, Valeria Mondelli, Maria Nettis, Naghmeh Nikkheslat, Carmine Pariante, Karen Randall, Julia Schubert, Luca Sforzini, Hannah Sheridan, Camilla Simmons, Nisha Singh, Federico Turkheimer, Vicky Van Loo, Mattia Veronese, Marta Vicente-Rodríguez, Toby Wood, Courtney Worrell, Zuzanna Zajkowska

Lundbeck

Brian Campbell, Jan Egebjerg, Hans Eriksson, Francois Gastambide, Karen Husted Adams, Ross Jeggo, Thomas Moeller, Bob Nelson, Niels Plath, Christian Thomsen, Jan Torleif Pederson, Stevin Zorn

NHS Greater Glasgow and Clyde

Catherine Deith, Scott Farmer, John McClean, Andrew McPherson, Nagore Penandes, Paul Scouller, Murray Sutherland

Oxford Health NHS Foundation Trust

Mary Jane Attenburrow, Jithen Benjamin, Helen Jones, Fran Mada, Akintayo Oladejo, Katy Smith

Pfizer

Rita Balice-Gordon, Brendon Binneman, James Duerr, Terence Fullerton, Veeru Goli, Zoe Hughes, Justin Piro, Tarek Samad, Jonathan Sporn

Sussex Partnership NHS Foundation Trust

Liz Hoskins, Charmaine Kohn, Lauren Wilcock

University of Cambridge

Franklin Aigbirhio, Junaid Bhatti, Ed Bullmore, Sam Chamberlain, Marta Correia, Anna Crofts, Tim Fryer, Martin Graves, Alex Hatton, Manfred Kitzbichler, Mary-Ellen Lynall, Christina Maurice, Ciara O'Donnell, Linda Pointon, Peter St George Hyslop, Lorinda Turner, Petra Vertes, Barry Widmer, Guy Williams

University of Glasgow

Jonathan Cavanagh, Alison McColl, Robin Shaw

University of Groningen

Erik Boddeke

University of Oxford

Alison Baird, Stuart Clare, Phil Cowen, I-Shu (Dante) Huang, Sam Hurley, Simon Lovestone, Alejo Nevado-Holgado, Elena Ribe, Anviti Vyas, Laura Winchester

University of Southampton

Madeleine Cleal, Diego Gomez-Nicola, Renzo Mancuso, Hugh Perry

University of Sussex

Mara Cercignani, Charlotte Clarke, Alessandro Colasanti, Neil Harrison, Rosemary Murray

University of Texas

Jason O'Connor

University of Toronto

Howard Mount

**Supplemental Figure 1. Iba1, TSPO and GFAP immunofluorescence in the intracranial (ic) vehicle- and LPS- treated rats.**

(a) Whole brain section from Iba1 (red) and GFAP (green)-immunostained ipsilateral and contralateral striatum of ic LPS-treated rats. (b) Magnifications of contralateral and ipsilateral side of the brain. White arrowheads: ramified microglia (Iba1) or filamentous astrocytes (GFAP). Clear arrowheads: amoeboid/activated microglia (Iba1) or reactive astrocytes (GFAP). (c) Confocal photomicrographs from Iba1 (green) and TSPO (red)-immunostained ipsilateral and contralateral striatum of ic LPS- and vehicle- treated rats. Nuclear counterstaining was performed using DAPI (blue). Scale bar = 2 mm, scale bar magnification=20 μm.

| **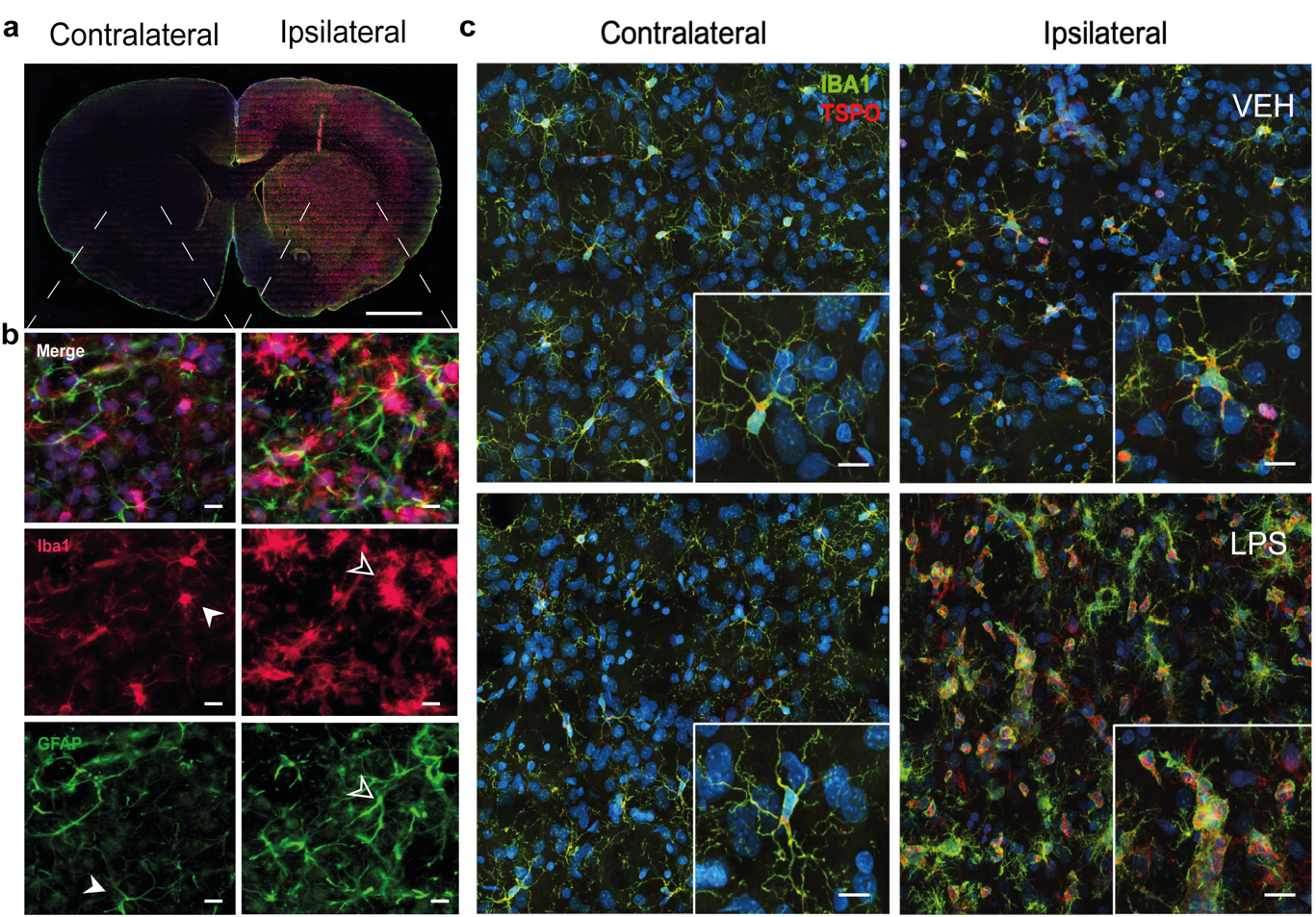** |
| --- |

| **Supplemental Figure 2. Inflammatory reaction 4 days after intrastriatal LPS injection.** mRNA genes expression 4 days after LPS administration in brain homogenates. |
| --- |
| **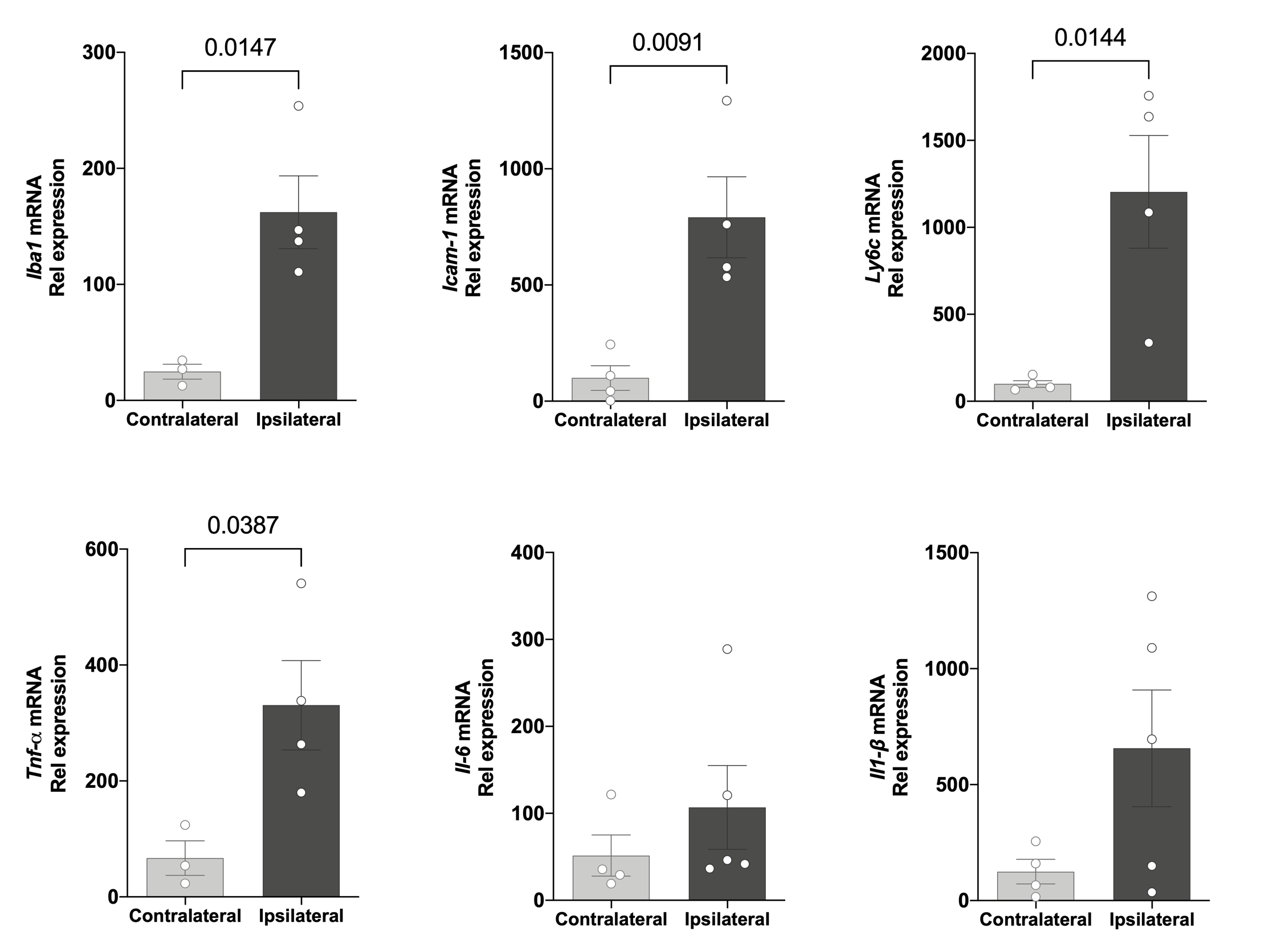** |

**Supplemental Figure 3. AUC of TACs for (a) blood and plasma radioactivity over the 60 min scan, and (b) injected dose per weight of animal (ID/g).**

| 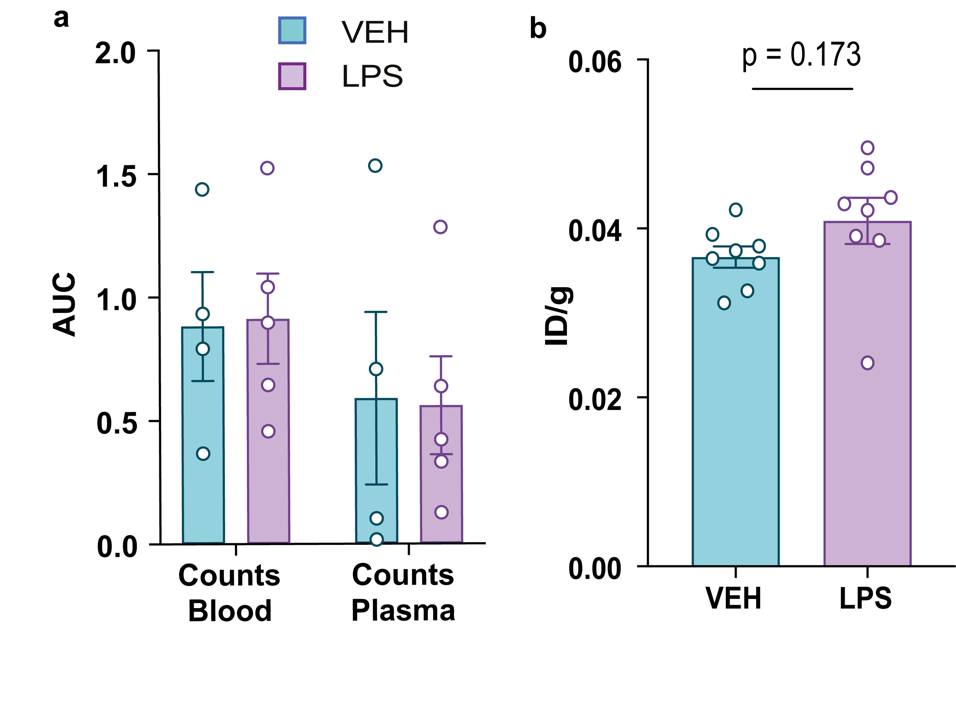 |
| --- |

| **Supplemental Figure 4. mRNA expression of different inflammatory genes in ip LPS- and vehicle- treated rats in brain homogenates.** |
| --- |
| **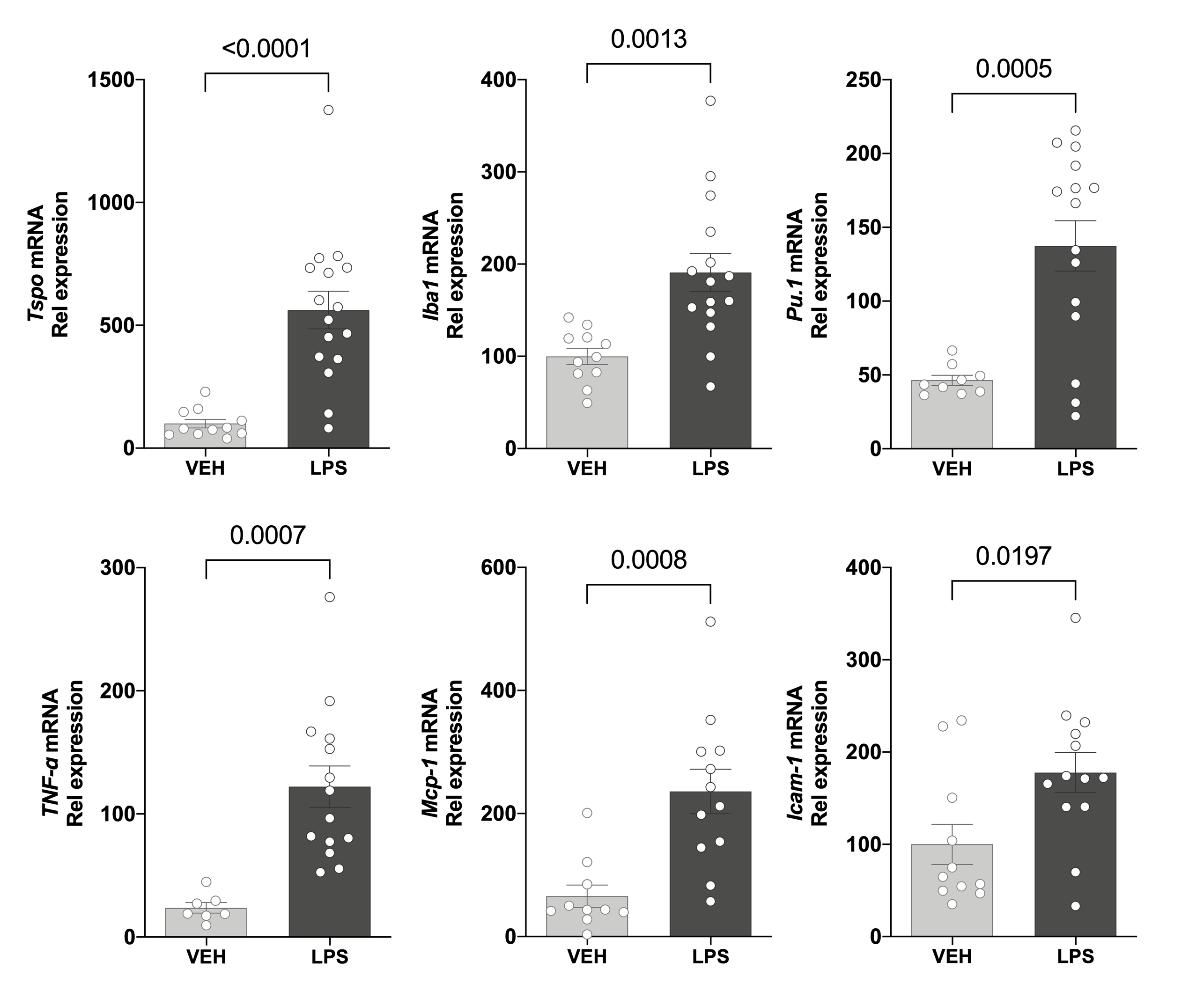** |

### Supplemental Table 1. Primary and secondary antibodies used for immunofluorescence

| \| \| Primary Antibodies \| Dilution \| Supplier \| Catalog number \| \| \| --- \| --- \| --- \| --- \| --- \| \| Goat anti-Iba1 \| 1:1000 \| Abcam \| #ab5076 \| \| Rabbit anti-TSPO \| 1:2000 \| Non-commercial \| NP155 \| \| Chicken anti-GFAP \| 1:1000 \| Abcam \| #ab4674 \| \| Chicken anti-NeuN \| 1:200 \| Synaptic Systems \| #266006 \| \| Goat anti-CD31 \| 1:50 \| R&d System \| #AF3628 \| \| Secondary Antibodies \| **Dilution** \| **Supplier** \| **Catalog number** \| \| Alexa Fluor 568 donkey anti-rabbit IgG \| 1:1000 \| Thermo Fisher \| #A10042 \| \| Alexa Fluor 488 donkey anti-chicken IgY \| 1:600 \| Jackson Immuno \| #703-545-155 \| \| Alexa-Fluor 647 donkey anti-goat IgG \| 1:600 \| Jackson Immuno \| #705-605-147 \| \| \| --- \| --- \| --- \| --- \| --- \| --- \| --- \| --- \| --- \| --- \| --- \| --- \| --- \| --- \| --- \| --- \| --- \| --- \| --- \| --- \| --- \| --- \| --- \| --- \| --- \| --- \| --- \| --- \| --- \| --- \| --- \| --- \| --- \| --- \| --- \| --- \| --- \| --- \| --- \| --- \| --- \| --- \| |
| --- | --- | --- | --- | --- | --- | --- | --- | --- | --- | --- | --- | --- | --- | --- | --- | --- | --- | --- | --- | --- | --- | --- | --- | --- | --- | --- | --- | --- | --- | --- | --- | --- | --- | --- | --- | --- | --- | --- | --- | --- | --- | --- |

### Supplemental Table 2. RNAscope probes used for RNAscope

| \| \| Probe Names \| Catalog Number \| Opal Fluor \| Catalog number \| \| --- \| --- \| --- \| --- \| \| RNAscope® Probe - Rn-Tspo \| 543531 \| Opal 520 \| Akoya Biosciences PNFP1487001KT \| \| \| RNAscope® Probe - Rn-Ccr2-C3 \| 478921-C2 \| Opal 570 \| Akoya Biosciences PNFP1488001KT \| \| \| RNAscope® Probe - Rn-Tmem119-C2 \| 526281-C3 \| Opal 690 \| Akoya Biosciences PNFP1497001KT \| \| \| \| --- \| --- \| --- \| --- \| --- \| --- \| --- \| --- \| --- \| --- \| --- \| --- \| --- \| --- \| --- \| --- \| --- \| --- \| --- \| --- \| |
| --- | --- | --- | --- | --- | --- | --- | --- | --- | --- | --- | --- | --- | --- | --- | --- | --- | --- | --- | --- | --- |

### Supplemental Table 3. [^18^F]DPA-714 SUV AUC values from all ROIs analysed following ic-LPS treatment. ** p < 0.01 vs. Contralateral

| \| **ROI** \| **Contralateral** \| **Ipsilateral** \| \| --- \| --- \| --- \| \| Olfactory \| 57.39 ± 3.59 \| 58.13 ± 4.05 \| \| Cortex \| 33.04 ± 2.84 \| 38.60 ± 4.12 \| \| Basal ganglia \| 26.29 ± 2.44 \| 37.61 ± 4.29** \| \| Corpus callosum \| 25.97 ± 2.25 \| 33.69 ± 3.65 \| \| Hippocampus \| 30.84 ± 2.38 \| 33.23 ± 3.71 \| \| Thalamus \| 25.86 ± 4.11 \| 31.93 ± 7.19 \| \| Amygdala \| 35.64 ± 3.17 \| 41.62 ± 2.39 \| \| Hypothalamus \| 37.93 ± 5.64 \| 43.41 ± 8.60 \| \| Midbrain \| 36.12 ± 3.39 \| 36.03 ± 3.23 \| \| Septal area \| 30.39 ± 3.99 \| 35.12 ± 5.01 \| \| Ventricles \| 31.31 ± 3.47 \| 38.26 ± 3.31 \| \| White matter \| 35.67 ± 4.08 \| 42.38 ± 4.30 \| \| Cerebellum \| 44.44 ± 2.32 \| 41.14 ± 3.22 \| |
| --- | --- | --- | --- | --- | --- | --- | --- | --- | --- | --- | --- | --- | --- | --- | --- | --- | --- | --- | --- | --- | --- | --- | --- | --- | --- | --- | --- | --- | --- | --- | --- | --- | --- | --- | --- | --- | --- | --- | --- | --- | --- | --- |
